# 120th Anniversary of the Kraepelinian Dichotomy of Psychiatric Disorders

**DOI:** 10.1007/s11920-019-1048-6

**Published:** 2019-07-01

**Authors:** Janusz K. Rybakowski

**Affiliations:** 1 Department of Adult Psychiatry, Poznan University of Medical Sciences, Szpitalna 27/33, 60-572 Poznan, Poland; 2 Department of Psychiatric Nursing, Poznan University of Medical Sciences, Poznan, Poland

**Keywords:** Kraepelin, Dichotomy, Schizophrenia, Mood disorder, Bipolar

## Abstract

**Purpose of Review:**

Emil Kraepelin, in 1899, proposed a dichotomy of psychiatric disorders into “dementia praecox,” further called schizophrenia, and “manisch-depressives Irresein,” now conceptualized as a bipolar disorder. The purpose of the review is to show both similarities and differences between disorders involved in this dichotomy, speaking for and against the idea.

**Recent Findings:**

On the molecular genetic side, there are data for both a genetic overlap and genetic differences between these two illnesses. Among pharmacological treatment, lithium, valproates, and carbamazepine present evidence for Kraepelinian dichotomy while atypical antipsychotics speak against this. The recent results for similarities and differences in the immune system, cognitive functions, and neurodevelopmental mechanisms have also been presented and discussed.

**Summary:**

As of 2019, the Kraepelinian dichotomy has been still partly valid although the results of recent clinical, neurobiological, and pharmacological studies provided a large number of data for an intermediate space between schizophrenia and bipolar disorder.

## Introduction

In 1899, a German psychiatrist, Emil Kraepelin (1856–1926), acting at that time as the Professor of psychiatry and director of the Psychiatric Clinic of the Heidelberg University, published the sixth edition of his textbook on psychiatry [1]. In this monograph, he made a fundamental dichotomous distinction of psychiatric disorders which paved the way for psychiatric diagnosis for the twentieth century and even beyond. On the basis of the course and outcome, the main aspect of which was a cognitive deterioration, he delineated two main groups of psychiatric disorders. The first, with chronic course and systematic cognitive deterioration, was called “dementia praecox.” The second, with the periodic course and a relative lack of cognitive deterioration, was called “Manisch-depressives Irresein” [2]. “Dementia praecox” was further conceptualized as “schizophrenia” by the Swiss psychiatrist Eugen Bleuler [3]. From the current perspective, “Manisch-depressives Irresein” has been now the closest to the concept of “bipolar mood disorder.” The aim of this review is to show both similarities and differences between disorders involved in this dichotomy, speaking for and against the idea.

### Schizoaffective Disorder

In the later period of his life, Kraepelin himself admitted that there is a proportion of patients where his classification is not possible to make [4]. However, it was not until 1933 when an American psychiatrist, Jacob Kasanin, coined a term of “schizoaffective psychosis” for describing a clinical phenomenon occupying a space between two dichotomously parted entities mentioned above [5]. With the advent of modern psychiatric classification in the last decades of the twentieth century, the schizoaffective psychosis found its place in both International Classification of Diseases (ICD) and Diagnostic and Statistical Manual of Mental Disorder (DSM). In the ICD-10, released in 1992, schizoaffective disorders, coded as F25, are included in the group of schizophrenia, schizotypal, and delusional disorders [6]. In the DSM-IV, released in 1994, schizoaffective disorder, coded as 295.7, was placed in the group of schizophrenia and other psychiatric disorders [7]. This was also retained in the DSM-5, introduced in 2013, in the group of schizophrenia spectrum and other psychotic disorders [8].

Some important studies on schizoaffective disorders appearing in the twenty-first century have not brought about any definite conclusions. In 2008, Brazilian authors performed a systematic review of 49 studies that compared schizoaffective disorders with schizophrenia or mood disorder. Evaluation of demographic characteristics, symptomatology, other clinical data, dexamethasone suppression test, neuroimage exams, response to treatment, evolution, and family morbidity indicated that schizoaffective disorder occupies an intermediate position between schizophrenia and mood disorders. The analysis indicated that schizoaffective disorder cannot be interpreted as an atypical form of schizophrenia or mood disorder and does not appear to represent either their co-morbidity or an independent mental disorder. The authors argued that schizoaffective disorder might constitute a heterogeneous group composed of both schizophrenia and mood disorder patients or a middle point of a continuum between these two entities [9].

Keshavan et al. in 2011 [10] devised a brief descriptive scale based on the type and relative proportions of psychotic and affective symptoms over the illness course and administered it to a series of 762 patients with psychotic disorders, including schizophrenia, schizoaffective, and psychotic bipolar disorder. The resulting Schizo-Bipolar Scale scores across these disorders showed neither a clear dichotomy nor a simple continuous distribution. While the majority of cases had ratings close to prototypic schizophrenia or bipolar disorder, a large group (45% of cases) fell on the continuum between these two prototypes. Kotov et al. in 2013 [11•] evaluated associations between symptom course and long-term outcome in 526 patients, for 4 years, and in 413 of them, for 10 years. They used nonlinear modeling to examine links between 4-year symptom variables (ratio of nonaffective psychosis to mood disturbance, duration of mania/hypomania, depression, and psychosis) and 10-year outcomes. Non-affective psychosis ratio exhibited a sharp discontinuity—10 days, or more of psychosis outside mood episodes predicted an 11-point decrement in Global Assessment of Functioning (GAF)—consistent with the Kraepelinian dichotomy. Duration of mania/hypomania showed two discontinuities demarcating three groups: mania absent, episodic mania, and chronic mania (manic/hypomanic > 1 year). The episodic group had a better outcome compared with the mania absent and chronic mania groups. Duration of depression and psychosis had linear associations with worse outcome. The authors suggest that their data support the Kraepelinian dichotomy, since in their study, a boundary between schizoaffective disorder and schizophrenia was not observed, which may cast doubt on schizoaffective diagnosis. They postulate that co-occurring schizophrenia and mood disorder may be coded as separate diagnoses.

### Continuum or Staging Concepts of Psychiatric Disorders

The Kraepelinian dichotomy of psychiatric disorders made a significant paradigm shift as far as a classification of psychiatric disorders was concerned. However, such a view was both preceded and succeeded by the theories postulating a continuum or staging among psychiatric disorders. The most influential concept of this kind in the nineteenth century was that of the German psychiatrist Wilhelm Griesinger (1817–1868). Griesinger argued that madness was the consequence of a single disease of the brain. According to his conception of the “unitary psychosis” (*Einheitspsychose*), the manifold symptoms of madness were not the result of different diseases but different stages of a single disease process [12]. Griesinger adapted the idea of the “unitary psychosis” from his mentor Ernst Albrecht von Zeller (1804–1877). According to Zeller, the continuum or staging of mental disorders begins with melancholia and further progressing to mania, paranoia, and dementia [13].

Furthermore, as the staging intensity of mental disorders in the nineteenth century is concerned, the credit should also be given to a Belgian psychiatrist, Joseph Guislain (1797–1860), who postulated the following staging of the severity of mental disorders (phrenalgie) (in French): manie-folie-stupidité-l’epilepsie-hallucinations-confusion-dementia [14]. Also, a German psychiatrist, Heinrich Neumann (1814–1888), can be mentioned here who staged “Irresein” (madness) beginning from Wahsinn (insanity) to Verwirrheit (confusion) and further Blődsinn (dementia) [15].

Probably, the most important continuum concept of mood disorders and schizophrenia in the twentieth century was that of the British psychiatrist Timothy Crow. According to him, the continuum extends from unipolar, through bipolar affective illness and schizoaffective psychosis, to typical schizophrenia, with increasing degrees of the neuropsychological defect. Crow tried to support this hypothesis with molecular genetic underpinnings which mostly have been not confirmed in subsequent research [16].

### Dichotomous Neurotransmitter Concepts of Schizophrenia and Mood Disorders

The dichotomy of psychiatric diagnosis has been reflected in neurobiological research performed in recent decades and studying neurotransmitter functions. Presently, there are two dichotomous neurotransmitter concepts related to schizophrenia and mood disorders. The bipolar dopaminergic theory has been elaborated in connection with both schizophrenia and bipolar disorder. The glutamatergic concept suggests a deficit of glutamatergic mechanisms, especially the N-methyl-D-aspartate (NMDA) receptors in schizophrenia and excess in depression.

Dichotomous dopaminergic theory of schizophrenia postulates an increase of dopaminergic function in subcortical areas, underlying positive (psychotic) symptoms, and a decrease of this function in cortical areas, connected with negative (deficit) symptoms. The direct confirmation of these has not been made until the late 1990s when the neuroimaging studies with dopaminergic markers became available. For instance, Breier et al. [17] in patients with exacerbation of schizophrenia demonstrated increased amphetamine-induced striatal dopamine release which correlated with the intensity of positive symptoms. As to cortical areas, Slifstein et al. [18] provided the evidence for a deficit in the capacity of dopamine release in the dorsolateral prefrontal cortex in schizophrenia which correlated with impairment of working memory connected with this structure. Recently, as the main mechanism of hyperdopaminergic abnormality in schizophrenia, the elevation in presynaptic dopaminergic function is postulated, which affects dopamine synthesis capacity, baseline synaptic dopamine levels, and dopamine release [19••]. Also, in recent years, many authors point to an important contribution of stress and environmental factors to increased dopaminergic activity in schizophrenia [20].

Dichotomous dopaminergic theory of bipolar disorder postulates an increase of dopaminergic function in mania and a decrease in depression. The origin of this concept could be traced to the catecholaminergic hypothesis of mood disorders formulated by Schildkraut more than half a century ago [21]. The dopaminergic theory of bipolar disorder was recently reviewed by Ashok et al. [22••]. The authors concluded that, nowadays, there had been the evidence for an elevation in striatal dopaminergic D2/D3 receptor availability as a basis for increased dopaminergic neurotransmission in mania, and in depression—increased striatal dopamine transporter (DAT) leading to reduced dopaminergic function.

The therapeutic action in a mania of antipsychotic drugs, both typical and atypical, blocking D2/D3 receptors has been well documented. However, in neuroimaging studies, increased dopaminergic activity was demonstrated only in psychotic mania [23]. The evidence for an antidopaminergic activity of other antimanic drugs such as lithium or valproate has been mostly obtained in experimental studies. Can et al. [24] observed that lithium treatment ameliorated mania phenotypes in mice by normalizing dopamine release in the nucleus accumbens. In the study of Landgraf et al. [25], valproate exerted a suppressive effect on disturbances of the circadian period resulting from hyperdopaminergic activity in mice.

The role of decreased dopaminergic activity in depression was reviewed by Dailly et al. [26]. The role of the dopamine system in depression has been implicated by both experimental and human studies. A dopamine deficiency in the mesolimbic pathways has been postulated, based on animal models of depression, with the special focus on such a deficiency connected with anhedonia. Clinical studies provided evidence of antidepressant action exerted by dopaminergic drugs. The main drug in this respect is bupropion, which inhibits dopamine reuptake by the dopamine transporter (DAT). A dopaminergic element (enhancing dopamine system function) can be observed in many antidepressant drugs, the examples of which being moclobemide and tianeptine. However, it has been indicated that antidepressant action can also be obtained with atypical antipsychotic drugs, blocking D2 receptors, which can be probably connected with other mechanisms [26]. Furthermore, the effect of stress condition on the dopaminergic system activity in mood disorders has been postulated [27].

The dichotomous glutamatergic theory of major psychiatric disorder suggests a hypoactivity of this system in schizophrenia and hyperactivity in depression. An abnormality of glutamatergic neurotransmission has been postulated in schizophrenia in the mid-1990s, and it was formulated as the NMDA receptor hypofunction [28]. Glutamatergic abnormality in schizophrenia has been confirmed by some molecular genetic studies in the early 2000s [29]. This prompted the attempts of using the inhibitors of so-called glycine site (a part of the NMDA receptor) for augmentation of antipsychotic drugs in the treatment of negative schizophrenic symptoms [30].

The concept of an increased NMDA activity in depression arose after showing a rapid antidepressant effect of the infusion of ketamine, an NMDA antagonist [31]. In our recent study, we demonstrated that ketamine augmentation rapidly improves depression scores in inpatients with treatment-resistant bipolar depression [32].

### Genetics of Schizophrenia and Bipolar Disorder

Recent genetic studies may speak both for and against the Kraepelinian dichotomy. A lot of research suggest a genetic overlap between schizophrenia and bipolar disorder. In 2009, Lichtenstein et al. [33] searching more than 2 million nuclear families in Sweden showed that first-degree relatives of probands with either schizophrenia or bipolar disorder were at increased risk of both these disorders. In the same year, the results of the genome-wide association study (GWAS) from the International Schizophrenia Consortium confirmed a substantial overlap of common polygenic variation between these two illnesses [34•].

The most detailed comparison of the genetic risk underlying schizophrenia and bipolar disorder was made by Ruderfer et al. [35]. They performed a combined GWAS of 19,779 schizophrenia and bipolar disorder cases versus 19,423 controls, in addition to a direct comparison GWAS of 7129 schizophrenia versus 9252 bipolar disorder cases. Apart from evidencing common genes for these two conditions, among them the most significant being the *CACNA1C* (calcium voltage-gated channel subunit alpha1 C) gene, they created a polygenic risk score for these two conditions. They found that the polygenic risk score of bipolar disorder can predict the clinical dimension of mania in schizophrenia. Surprisingly, in their study, they did not find a correlation between a polygenic risk score of schizophrenia and the clinical dimension of psychosis in bipolar disorder, although many clinical and previous genetic studies had suggested such a possibility.

It looks like a genetic difference between schizophrenia and bipolar illness may lie in the magnitude of the copy number variations (CNV) which are chromosomal micro-duplications or deletions. These abnormalities have also been evidenced in GWAS research. It was found that such genetic abnormalities are significantly more common in schizophrenia than in bipolar illness, thus making a meaningful distinction between these two disorders [36].

### Pharmacology of Schizophrenia and Bipolar Disorder

Antipsychotic drugs are the mainstay for the acute and long-term treatment of schizophrenia. All of them have also been efficacious for the treatment of mania. However, in bipolar disorder, so-called typical antipsychotics (e.g., haloperidol) can precipitate the occurrence of a depressive episode and do not prevent the affective recurrences. Whereas in the process of introduction to psychiatric therapy of so-called atypical antipsychotics such as clozapine, olanzapine, quetiapine, aripiprazole or risperidone, it has been noticed that they possess mood-stabilizing properties. The first such observation about clozapine was made by Zarate et al. in 1995 [37].

A definition of mood-stabilizer includes (1) reducing or ameliorating manic and/or depressive episodes, (2) preventing recurrent manic and/or depressive episodes, and (3) not inducing or worsen manic and/or depressive episodes. A proposal was made to divide mood stabilizers based on the chronology of their introduction in psychiatric therapy into the first generation (introduced in 1960–1970, such as lithium, valproates, and carbamazepine) and the second generation, introduced after Zarate et al.’s observation, to which atypical antipsychotics belong [38]. All atypical antipsychotic drugs exert both acute and prophylactic antimanic activity and some (e.g., quetiapine) also antidepressant activity [39•]. Therefore, the use of the atypical antipsychotic drugs in the acute treatment and prophylaxis of bipolar mood disorder can make evidence against Kraepelinian dichotomy.

On the other hand, the therapeutic activity of the “classical” mood-stabilizing drugs such as lithium, valproates, and carbamazepine in bipolar disorder and not in schizophrenia can make the argument for Kraepelinian dichotomy. The first clinical report on the use of lithium in contemporary psychiatry was made by the Australian psychiatrist John Cade, 70 years ago [40], revealing a significant antimanic effect of the drug. In 1963, the British psychiatrist Geoffrey Hartigan [41] was the first to report a beneficial effect of long-term lithium treatment on the prevention of manic and depressive recurrences in patients with affective disorders. Since then, the use of lithium has been the most proven long-term pharmacotherapy of bipolar disorder for the prophylaxis of affective episodes [42]. Among bipolar patients, there is a group called “excellent lithium responders” in which a maintenance lithium monotherapy can make the illness not existing [43] which may point to a precise therapeutic specificity of lithium in such patients. The percentage of this group amounts to 1/3 of bipolar patients [44], and their clinical characteristics may resemble a classical description of Kraepelinian “manisch-depressives Irresein” with a moderate number of the episodes and complete remission of symptoms between the episodes. On the other hand, lithium does not possess antipsychotic activity, and its prophylactic efficacy has been negatively correlated with a feature of cognitive disorganization, connected with a predisposition to psychosis [45]. Also, in a recent GWAS research, the polygenic score for schizophrenia was inversely correlated with lithium treatment response [46].

Anticonvulsants valproate and carbamazepine belonging to the first-generation mood-stabilizers exert significant antimanic and prophylactic effect in bipolar disorder but not antipsychotic activity in schizophrenia. Whether they may exert an antipsychotic action in psychotic mania has not been definitely established. Anticonvulsant lamotrigine, regarded as a second-generation mood stabilizer [38], exerts a significant antidepressant and prophylactic activity in bipolar disorder. However, the drug does not act therapeutically in mania and does not exert antipsychotic activity in schizophrenia [47]. Therefore, lithium and anticonvulsant mood stabilizers of the first and second generation can make evidence for Kraepelinian dichotomy.

### Immunology of Schizophrenia and Bipolar Disorder

Brown [48••] discusses an immunological aspect of the Kraepelinian dichotomy from the perspective of prenatal infections and immunologic insults. In his previous review, it was demonstrated that maternal influenza had been associated with schizophrenia in their offspring [49]. Several studies have found such an association also with bipolar disorder; however, the most significant study of Canetta et al. [50] showed that maternal serological influenza exposure was related to a significant fivefold greater risk of bipolar disorder with psychotic features suggesting that, among offspring, maternal influenza may increase the risk of psychosis, rather than schizophrenia per se.

The studies of other prenatal infections suggest that they may have an association with an increased risk for schizophrenia. However, such a connection with bipolar disorder is doubtful. This pertains to the infections with *Toxoplasma gondii* as well to herpes simplex virus type 2 (HSV-2) [48••].

The possible differences in the indexes of the immune system in schizophrenia and bipolar illness can also be traced in adult age. Karpiński et al. [51] compared leukocyte proportions in these two illnesses and found the reduced number of peripheral natural killer (NK) cells in schizophrenia but not in bipolar disorder. In schizophrenia, there were no significant differences in the number of NK cells between periods of exacerbation and remission. Recently, Tanaka et al. [52] measured plasma levels of antibodies and innate immune markers in patients with schizophrenia and bipolar disorder and found an increase in soluble CD14 in both disorders, and in bipolar disorder, also, an increase in C-reactive protein, IgM class antibodies against cytomegalovirus (CMV), and IgG class antibodies against HSV-2 as well as a negative relationship between IgG antibodies against CMV and scores for cognitive functions.

### Cognitive Functions in Schizophrenia and Bipolar Disorder

Because the phenomenon further named “schizophrenia” was termed by Kraepelin “dementia praecox,” the cognitive functions which made a criterion for such a distinction between two illnesses have recently been an increasing subject of interest. In 1987, Keefe et al. [53] proposed a distinction between Kraepelinian and non-Kraepelinian schizophrenia based on a degree of severe and debilitating course of the illness, typical of Kraepelin’s description of “dementia praecox.” Sixteen years later, in the neuropsychological study of these two kinds of the illness, Roy et al. [54] demonstrated that Kraepelinian schizophrenia is characterized by significantly more impaired performance on a variety of neuropsychological tests.

Notwithstanding the difference of cognitive dysfunctions within schizophrenia subjects, it seems that a degree of cognitive impairment can differentiate schizophrenia and bipolar disorder. By contrast to schizophrenia, which is characterized by a broad global cognitive impairment that precedes the onset of the disease, this is not the case in bipolar disorder. In a Danish draft board population, the mean educational level was similar between those hospitalized later for bipolar disorder and control subjects [55]. In Swedish research, individuals with excellent school performance had a nearly fourfold increased risk of later bipolar disorder compared with those with average grades [56]. Vreeker et al. [57•] investigated intelligence and educational performance in patients with schizophrenia and bipolar disorder as well as in their relatives and showed that high educational performance is a distinctive feature of bipolar disorder. On the other hand, it is generally agreed that after the onset of illness, patients with both schizophrenia and bipolar disorder seem to suffer a further decline in cognitive function, with the magnitude of the impairment greater in schizophrenia than in bipolar disorder [58•].

In our research, using the Wisconsin Card Sorting Test for assessing of cognitive efficiency, we demonstrated that the performance on this test of patients with bipolar disorder is worse than healthy controls but significantly better than patients with schizophrenia [59]. In another study, we showed that in “excellent lithium responders” even with long duration of bipolar illness, the performance on cognitive tests was not different than that of healthy control subjects [60].

Recently, Jimenez-Lopez et al. [61] studied neurocognitive functions in schizophrenia and bipolar patients. In both groups of patients, the performance was significantly worse than in control subjects. However, in bipolar patients, it was more impaired in subjects with psychotic symptoms. This may again suggest an association between psychotic bipolar disorder and schizophrenia.

### Neurodevelopmental Pathogenesis of Schizophrenia and Bipolar Disorder

The similarities and differences between schizophrenia and bipolar disorders can also be conceptualized within the framework of the neurodevelopmental pathogenesis of psychiatric disorders. Demjaha et al. [62•] proposed a model where, on a shared genetic liability for both disorders, patients with schizophrenia have been subject to more additional genetic and environmental factors that impair development. Furthermore, some environmental factors can exert a stronger impact on the development of schizophrenia than bipolar disorder. As a result of this, most schizophrenia patients at the first onset of illness show neuroanatomical changes and cognitive deficits which is not the case in patients with bipolar disorder. Premorbid adjustment of schizophrenia patients is significantly worse than that of control subject while such an impairment in bipolar patients has not been shown [63].

The additional factor already mentioned, impairing development in schizophrenia, is an excess in CNVs which appears to be common in schizophrenia, autism, and epilepsy. This may be responsible for the subtle neuromotor, cognitive, and behavioral impairment seen in schizophrenia patients already in their childhood years. Another factor, distinctive for schizophrenia, may involve obstetric complications, in a majority of cases resulting in hypoxic damage to the hippocampus. Further stresses in adolescence or early adulthood can cause overactivity of the hypothalamic-pituitary-adrenal axis and hypercortisolemia which induces further damage to the hippocampus. Such a “second hit” to the developmentally compromised hippocampus can lead to dopamine system dysregulation what was shown in an animal model of schizophrenia [64].

Among other environmental factors influencing neurodevelopment, urbanicity has been repeatedly associated with increased incidence of schizophrenia [65] while such a connection with bipolar disorder has not been obtained [66]. Also, such a phenomenon as migration has been an important risk factor for schizophrenia [67], while there is no conclusive evidence for an increase of bipolar disorder associated with migration [68].

As a result of more impaired neurodevelopment, structural changes of the brain in patients with first onset illness such as lateral ventricular enlargement and gray matter abnormalities are much more marked in schizophrenia than in bipolar illness [62].

## Discussion

Following presentations of neurotransmitter evidence, genetics, pharmacology, immunology, cognitive function, and neurodevelopment pathogenesis, a picture emerges where some foundations of the Kreapelinian dichotomy on its 120th birthday are still valid while some have been questioned.

Dichotomous dopaminergic and glutamatergic hypotheses of schizophrenia and mood disorders may fit into the Kraepelinian dichotomy of these disorders. Also, the therapeutic effect of typical antipsychotics, antidepressants, and classic mood stabilizers in schizophrenia and mood disorders may speak for it. However, a real breach in this picture has appeared after so-called “atypical” antipsychotics have been introduced into psychiatric treatment, and a majority of these drugs have been found to possess mood-stabilizing properties such as an ability of prevention of bipolar mood episodes. Furthermore, some of them, such as quetiapine and lurasidone have been demonstrated to exert a strong antidepressant action in bipolar depression [69, 70].

Recent genetic studies point to a substantial genetic overlap between schizophrenia and bipolar disorder. However, within the genome, a specific polygenic risk score can be delineated for each of these conditions. Furthermore, the number of CNV can be a discriminating factor, being significantly higher in schizophrenia. Interestingly, in recent years, the observed diagnostic difficulties between these illnesses and borderline personality disorder have been substantiated by showing a genetic overlap [71] what was also the case with bipolar disorder and attention-deficit/hyperactivity disorder [72].

Among immunological findings, most of them point to relatively bigger changes in this respect in schizophrenia than in bipolar disorder. Interestingly, it is schizophrenia and psychotic (but not non-psychotic) bipolar disorder where influenza exposure in pregnancy has been related to a significantly greater risk of the illness. Schizophrenia and bipolar illness may differ in cognitive status (better in bipolar disorder) preceding the onset of the illness. However, after the onset of illness, the differences in cognition (worse in schizophrenia) are more quantitative than qualitative nature. Again, psychotic bipolar disorder in this respect is more akin to schizophrenia. Finally, based on analyses related to neurodevelopment, patients with schizophrenia can be subject to more additional genetic and environmental insults that impair development compared with bipolar disorder. All these findings can confirm both the commonality in the background between these two disorders and the increased abnormalities in many areas in schizophrenia than in bipolar illness.

## Conclusions

One-hundred and twenty years of presence of Kraepelinian dichotomy of psychiatric disorders has exerted an enormous impact on psychiatric diagnosis and treatment. It can be stated that, as of 2019, this great idea has been still partly valid.

It is known that Kraepelin himself assumed a possibility of not so definite boundaries between “dementia praecox” and “manisch-depressives Irresein” in many patients, and a meaningful contribution to these doubts was a concept of schizoaffective disorder in the 1930s [5]. However, it was not until the 1990s when a significant confirmation for an intermediate space between schizophrenia and bipolar disorder has been obtained by the psychopharmacological and genetic studies. The main pharmacological argument for this is mood-stabilizing properties of the atypical antipsychotic drugs while, for the other, a shared molecular-genetic make-up of schizophrenia and bipolar disorder.
